# Artificial Intelligence in Gallbladder Imaging: A Rapid Evidence Review and Exploratory Meta-Analysis of Diagnostic Performance and Reader Assistance

**DOI:** 10.7759/cureus.114379

**Published:** 2026-08-11

**Authors:** Saravanasingh Karan Chand Mohan Singh, Jayalakshmi J, Ethel Shiny, Nithyamala I, Suguna Mani, Suresh K

**Affiliations:** 1 General Medicine, National Institute of Siddha, Chennai, IND; 2 Siddha Pharmacology, National Institute of Siddha, Chennai, IND; 3 Siddha Pathology, National Institute of Siddha, Chennai, IND; 4 Siddha Pediatrics, National Institute of Siddha, Chennai, IND

**Keywords:** artificial intelligence, gallbladder diseases, prisma statement, quadas-2, radiomics, systematic review and meta-analysis

## Abstract

Artificial intelligence (AI) is increasingly being investigated for gallbladder imaging, but its clinical diagnostic performance, added value over conventional interpretation, and generalizability remain uncertain. We conducted a rapid evidence review with exploratory meta-analysis using a previously published scoping review of 16 ultrasound studies as the initial evidence anchor; 12 reports met the present eligibility criteria. A targeted PubMed search through June 2026 was supplemented by publisher-website screening, reference-list review, and backward and forward citation tracking. Because Embase, Scopus, Web of Science, and the Cochrane Library were not independently searched, the evidence-identification strategy was not intended to be comprehensive. The included studies were classified into two evidence strata: Stratum A, clinically referenced diagnostic-accuracy studies evaluated against histopathology or another clearly defined patient- or lesion-level clinical reference standard, and Stratum B, technical/non-clinical image-classification studies based primarily on image or dataset labels. Seventeen peer-reviewed reports were included: 15 clinically referenced studies and two technical/non-clinical studies.

Across gallbladder-polyp studies, reported performance varied according to target definition and validation design. Independent or temporal validation generally produced more conservative estimates than internal validation, supporting the need for external testing before clinical implementation. Only two independent or temporal cohorts provided reconstructable 2×2 data for neoplastic-polyp classification. Individual-study sensitivity ranged from 74.3% to 78.6%, while specificity ranged from 81.8% to 92.1%. Because only two studies were available, formal bivariate random-effects or hierarchical summary receiver operating characteristic synthesis was not considered reliable. Separate random-effects summaries of sensitivity (76.1%; 95% confidence interval (CI), 64.1%-85.1%) and specificity (87.8%; 95% CI, 73.9%-94.8%) were therefore presented strictly as descriptive exploratory estimates rather than conventional joint diagnostic test-accuracy meta-analysis estimates. The exploratory diagnostic odds ratio was 24.0 (95% CI, 10.1-57.1). Three reader-assistance studies reported absolute improvements in accuracy ranging from 6.6 to 15.1 percentage points with AI support.

Because paired case-level and reader-level variance data were unavailable, these findings were summarized descriptively and were not meta-analyzed. Gallbladder-cancer studies were too heterogeneous for quantitative pooling, while the two technical studies demonstrated image-classification feasibility but did not provide equivalent evidence of patient-level clinical diagnostic accuracy. AI shows potential for gallbladder-polyp characterization and reader support, particularly for less-experienced clinicians. However, confidence remains limited by retrospective and surgery-enriched populations, small independent validation cohorts, heterogeneous validation methods, and the targeted rather than comprehensive literature search. Broader evidence synthesis and prospective multicenter clinical validation are required before routine or autonomous clinical implementation.

## Introduction and background

Gallbladder disease includes a wide range of benign and malignant conditions, such as cholelithiasis, cholecystitis, gallbladder polyps, and gallbladder carcinoma [1]. Transabdominal ultrasonography (TAUS) is usually the first-line imaging method because it is widely available, does not use ionizing radiation, and can identify gallstones, gallbladder wall changes, and polypoid lesions. When TAUS is inconclusive or malignancy is suspected, endoscopic ultrasonography (EUS), contrast-enhanced ultrasonography (CEUS), computed tomography (CT), and magnetic resonance imaging (MRI) may provide additional diagnostic information [1-4]. One of the most important clinical challenges is distinguishing non-neoplastic cholesterol polyps from neoplastic lesions, including adenomas and carcinoma. This distinction matters because decisions about surveillance or cholecystectomy depend partly on the estimated risk of malignancy [2,3]. Histopathological examination after cholecystectomy is commonly considered the definitive reference standard. However, studies based mainly on surgical cohorts may include a disproportionate number of larger or more suspicious lesions and may therefore not fully reflect the wider spectrum of patients seen in routine clinical practice. Early gallbladder carcinoma can also be difficult to recognize because it may resemble benign wall thickening, chronic inflammation, or other gallbladder abnormalities on conventional imaging [2-4]. Artificial intelligence (AI), including machine learning (ML), radiomics, and deep learning (DL), is increasingly being studied as a tool to support gallbladder imaging. These approaches can detect complex textural, morphological, vascular, and higher-order imaging features that may not always be recognized consistently by visual interpretation alone. AI may therefore be used as an image classifier, triage tool, or clinical decision-support system (CDSS) and may help improve diagnostic consistency, particularly for less-experienced readers [5]. However, strong algorithmic performance does not automatically mean that an AI system will provide meaningful clinical benefit. Its usefulness depends on the quality of the reference standard, appropriate patient-level separation of development and validation datasets, independent or external validation, generalizability across different patient populations and imaging platforms, and the way clinicians interact with AI outputs. Human-AI collaboration may also introduce automation bias, in which clinicians place excessive confidence in an incorrect AI recommendation. For this reason, AI-assisted interpretation should be evaluated not only by measures such as sensitivity, specificity, and area under the receiver operating characteristic curve (AUC) but also by its influence on reader behavior, clinician-AI disagreement, and clinical decision-making. A 2024 scoping review of AI applications in gallbladder ultrasonography identified 16 studies published through December 2023 [5]. Most studies focused on gallbladder polyps and gallbladder carcinoma, while relatively little evidence was available for inflammatory gallbladder disorders. The literature was largely retrospective and depended heavily on internal validation, raising concerns about whether the reported performance would be reproduced in routine clinical settings. More recent studies have expanded the field to include EUS video analysis, dual-mode ultrasonography, CT-based models, and prospective, temporal, or independent validation cohorts.

Despite this growing evidence base, important uncertainties remain. The reported performance differs according to the target disease, imaging modality, patient selection, reference standard, data-splitting strategy, and validation method. Many studies still rely on retrospective or surgery-enriched cohorts, and robust external validation remains uncommon. In addition, some investigations evaluate AI using image-level or curated dataset labels rather than clearly defined patient-level clinical reference standards. Such technical image-classification evidence should therefore not be interpreted as equivalent to clinical diagnostic test accuracy (DTA) evidence because the reference standard and unit of analysis are fundamentally different. Accordingly, this rapid systematic review and exploratory meta-analysis aimed to evaluate the diagnostic performance and generalizability of AI in adult gallbladder imaging, with particular attention to validation strategy, target disease, imaging modality, AI-assisted reader performance, and barriers to clinical implementation. The review also considers methodological issues related to human-AI interaction, including automation bias, and identifies priorities for future prospective clinical validation.

## Review

Methodology

Review Design and Reporting

This study was conducted as a rapid evidence review with exploratory meta-analysis of published studies evaluating artificial intelligence in gallbladder imaging. A previously published scoping review was used as the initial evidence anchor, followed by a targeted PubMed update and supplementary citation-based searching. The evidence-identification process was therefore designed as a rapid, targeted review rather than a de novo comprehensive multi-database systematic search. Reporting was guided by Preferred Reporting Items for Systematic Reviews and Meta-Analyses (PRISMA) 2020 and, where applicable, the PRISMA extension for diagnostic test accuracy (PRISMA-DTA) principles. The protocol was not prospectively registered [6,7]. Screening, data extraction, and risk-of-bias assessment were not performed independently in duplicate, and these features were considered methodological limitations. These limitations are already acknowledged.

Review Question and Eligibility Criteria

The review question was structured according to the Participants, Index test, Reference standard, and Diagnosis/target condition framework. Eligible participants were adults with suspected, imaging-detected, or histopathologically confirmed acquired gallbladder disease or gallbladder lesions. Eligible index tests included artificial intelligence, machine learning, deep learning, radiomics, computer-aided diagnosis, and AI-assisted reader interpretation applied to transabdominal ultrasonography, endoscopic ultrasonography, contrast-enhanced ultrasonography, computed tomography, magnetic resonance imaging, magnetic resonance cholangiopancreatography, nuclear imaging, or multimodal imaging. Histopathology was the preferred clinical reference standard. Where tissue confirmation was unavailable, clearly defined expert-consensus diagnoses or clinical follow-up standards were acceptedwhen they represented a meaningful patient- or lesion-level clinical diagnosis. Target conditions included neoplastic versus non-neoplastic gallbladder polyps, malignant versus benign gallbladder lesions, gallbladder carcinoma, gallstones, and other acquired gallbladder disorders. Comparators included unaided radiologists, sonologists, or endoscopists, conventional imaging criteria, and clinical prediction models. These eligibility concepts are already present in the submitted manuscript. Peer-reviewed original studies were eligible when they reported clinically interpretable diagnostic or classification outcomes, including sensitivity, specificity, accuracy, AUROC, diagnostic odds ratio, or change in reader performance. Reviews, editorials, commentaries, conference abstracts without sufficient results, pediatric congenital biliary disorders, prognostic studies without a diagnostic imaging focus, segmentation-only investigations, and studies limited exclusively to non-diagnostic image-processing outcomes were excluded.

Evidence-Stratum Classification

To account explicitly for differences in reference standards and units of analysis, the included studies were classified into two prespecified evidence strata.

Stratum A - clinically referenced diagnostic-accuracy studies: studies in which AI-based imaging predictions were evaluated against histopathology or another clearly defined patient- or lesion-level clinical reference standard.

Stratum B - technical/non-clinical image-classification studies: studies in which model performance was evaluated primarily against pre-existing image labels, dataset labels, or similar non-clinical annotations, particularly when image-level rather than patient-level diagnosis constituted the principal unit of analysis.

Stratum B studies were retained to characterize technical AI development but were synthesized separately and were not considered equivalent to clinically referenced diagnostic-accuracy evidence. Only eligible Stratum A studies contributed to pooled clinical sensitivity and specificity estimates.

Information Sources and Search Strategy

A previously published scoping review of artificial intelligence applications in ultrasonographic assessment of gallbladder disease was used as the initial evidence anchor. The review covered publications from January 2003 through December 2023 [5]. All potentially relevant reports identified in that review, together with studies cited in its reference list, were reassessed against the eligibility criteria of the present rapid evidence review. To identify more recent or additional eligible studies, a targeted literature update was conducted in PubMed/MEDLINE. The search combined concepts related to gallbladder disease, artificial intelligence, machine learning, diagnostic imaging, and diagnostic classification. PubMed/MEDLINE was the only bibliographic database independently searched for the present update. Embase, Scopus, Web of Science, and the Cochrane Library were not independently searched. Supplementary study identification included screening of publisher websites, reference lists of eligible studies and relevant reviews, and backward and forward citation tracking. These supplementary approaches were used to broaden retrieval and identify potentially relevant reports not captured by the targeted PubMed search; however, they were not intended to replace a comprehensive multi-database systematic search. Accordingly, the evidence-identification strategy was designed as a rapid and targeted update of the available literature. It should therefore not be interpreted as providing an exhaustive retrieval of all potentially eligible studies, and relevant reports indexed exclusively in databases not searched may have been missed.

Duplicate Handling and Study Selection

Records identified through the targeted update and supplementary searching were assessed for duplication using digital object identifier (DOI), PubMed identifier, article title, author names, publication year, and study population. Exact duplicate citations were removed before eligibility assessment. Potentially overlapping publications were reviewed at the full-text level. When multiple reports used the same or substantially overlapping study population, the most complete peer-reviewed report was retained for the relevant analysis unless another publication provided a distinct eligible outcome. Because the evidence-identification process relied partly on a previously published evidence map and complete historical record-level search logs were not available for all supplementary sources, some identification, screening, and duplicate-removal counts could not be reconstructed reliably. Such values were reported as NR (not recorded) in the PRISMA flow diagram rather than retrospectively estimated. The current figure already uses NR for unavailable historical counts.

Data Extraction

Eligible studies underwent standardized data extraction. Extracted variables included country, study design, number of centers, sample size, target condition, imaging modality, AI or machine-learning model, comparator, reference standard, evidence stratum, validation method, accuracy, sensitivity, specificity, and area under the receiver operating characteristic curve.

When full 2×2 contingency data were reported, the published values were used directly. When a study reported only accuracy and a corresponding denominator, the reported proportion and sample size were used for the exploratory accuracy synthesis. Integer contingency-table cells were not reconstructed from rounded accuracy estimates. 

Risk-of-Bias and Applicability Assessment

Risk of bias was assessed using a review-specific adaptation of Quality Assessment of Diagnostic Accuracy Studies (QUADAS-2) [8]. The four standard risk-of-bias domains - patient selection, index test, reference standard, and flow and timing - and the three standard applicability domains were retained. Additional AI-specific signaling considerations included patient-level versus image-level data splitting, independence of training and testing datasets, handling of multiple images from the same patient, prespecification of decision thresholds, exclusion of technically difficult cases, external validation, and potential data leakage. Each domain was categorized as low, high, or unclear risk or concern. The adapted framework was used as a review-specific extension and was not considered a separately validated risk-of-bias instrument. Stratum B technical/non-clinical studies were interpreted with particular caution because image-level splitting, dataset labels, patient overlap, and technical class balancing can limit applicability to patient-level clinical diagnosis.

Statistical Analysis

Quantitative synthesis was restricted according to target condition, clinical reference standard, validation design, and availability of reconstructable 2×2 diagnostic data. The main diagnostic analysis focused on AI classification of neoplastic versus non-neoplastic gallbladder polyps in clinically referenced independent or temporal patient-level test cohorts. Because only two eligible cohorts provided reconstructable 2×2 data, formal joint diagnostic test-accuracy synthesis using a bivariate random-effects or hierarchical summary receiver operating characteristic (HSROC) model was not considered sufficiently reliable. Such models are generally preferred because they jointly account for sensitivity and specificity, including their within-study correlation and threshold-related trade-off, but estimation of the additional covariance and heterogeneity parameters would be unstable with only two studies. Accordingly, the individual-study 2×2 results were treated as the primary diagnostic evidence. Separate random-effects summaries of sensitivity and specificity were calculated only as descriptive exploratory estimates. Sensitivity and specificity were analyzed on the logit scale because they are proportions bounded between 0 and 1; the transformation permits pooling on an unbounded scale and ensures that back-transformed estimates remain within the clinically meaningful range. Summary estimates and 95% confidence intervals were subsequently back-transformed and reported as proportions or percentages. Random-effects models were used because genuine between-study variation was anticipated owing to differences in patient spectrum, imaging acquisition, AI architecture, decision thresholds, and validation design. These models therefore incorporated both within-study sampling uncertainty and between-study heterogeneity. However, because sensitivity and specificity were summarized separately, these estimates do not account for their within-study correlation or threshold relationship and should not be interpreted as conventional joint DTA meta-analysis estimates. A diagnostic odds ratio (DOR) was calculated only as an exploratory secondary measure of overall discrimination. The DOR combines information from sensitivity and specificity, with a value of 1 indicating no discriminatory ability and larger values indicating greater discrimination. Because the DOR does not preserve the separate clinical interpretation of sensitivity and specificity or explicitly reflect threshold trade-offs, it was not considered a primary outcome. All summary estimates were therefore regarded as exploratory, provisional, and hypothesis-generating. Heterogeneity was summarized using I² and τ².

Results

Study Selection

**Figure 1:**
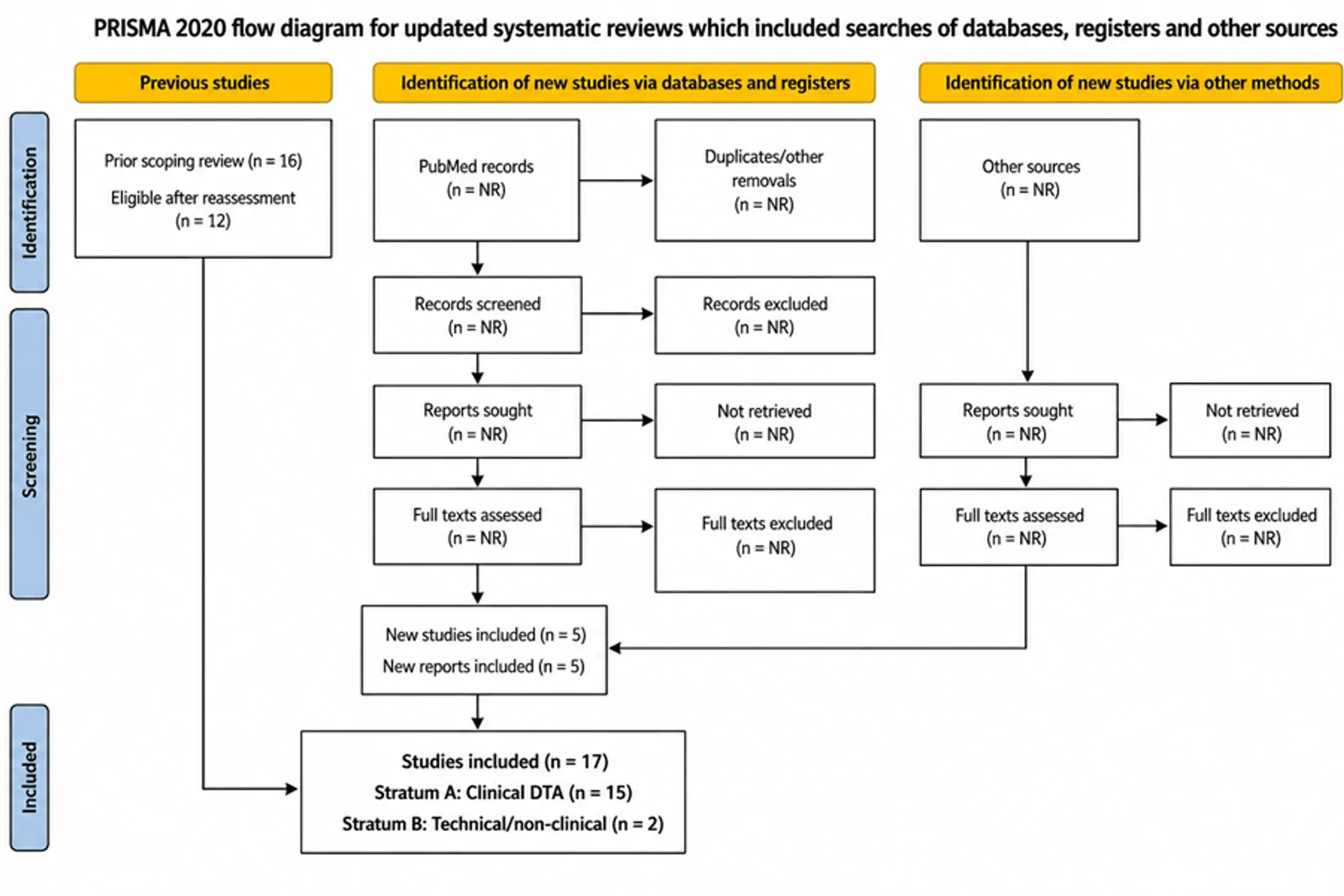
Study Identification and Inclusion Pathway for the Rapid Evidence Review The previous scoping review contained 16 reports, of which 12 met the present eligibility criteria. Five additional reports were identified through the targeted update and supplementary searching, resulting in 17 included reports. Complete historical record-level screening and deduplication counts were unavailable and were therefore not retrospectively estimated. Adapted from Page et al. [6].

The anchor scoping review contained 16 reports. Twelve met the present eligibility criteria; four were excluded because they addressed pediatric biliary atresia or another congenital disorder, were segmentation-only studies, or represented duplicate or preprint reports. The targeted update identified five additional eligible reports. Seventeen peer-reviewed reports were included overall. Of these, 15 were classified as clinically referenced diagnostic-accuracy studies (Stratum A), whereas two were classified as technical/non-clinical image-classification studies (Stratum B). The two technical studies used image- or dataset-label reference standards and were therefore analyzed descriptively rather than considered equivalent to clinically referenced DTA evidence. Eight gallbladder-polyp studies contributed to the exploratory accuracy synthesis, two independent or temporal test cohorts contributed reconstructable 2×2 data, and three studies directly compared AI-assisted with unaided reader interpretation. Figure 1 shows the PRISMA 2020 flow diagram for the anchor scoping review and targeted update search.

Study Characteristics

Twelve reports primarily evaluated gallbladder polyps or masses, three focused on gallbladder carcinoma, and two evaluated gallstones or multiclass disease detection. The evidence was dominated by retrospective surgical cohorts and internal validation. Prospective, external, temporal, or independently tested cohorts were reported in only a minority of studies. The 17 included reports comprised 15 clinically referenced diagnostic-accuracy studies and two technical/non-clinical image-classification studies. Their characteristics are presented in Table 1, with evidence stratum explicitly identified to distinguish clinical DTA evidence from technical image-classification evidence [9-25]. 

**Table 1:** Summary of the characteristics of the 17 included studies according to study setting, design and sample size, imaging modality, target condition, AI model or comparator, reference standard, validation strategy, and evidence stratum. Studies were classified as Stratum A (clinical DTA) when AI performance was evaluated against a clinical or pathological reference standard, and Stratum B (technical/non-clinical) when evaluation relied mainly on image or dataset labels. Stratum B studies were analyzed separately and were not considered equivalent to clinical diagnostic-accuracy evidence. AI: artificial intelligence; AUC: area under the receiver operating characteristic curve; CAD: computer-aided diagnosis; CEUS: contrast-enhanced ultrasonography; CNN: convolutional neural network; CT: computed tomography; CV: cross-validation; DL: deep learning; DTA: diagnostic test accuracy; DL-DSS: deep learning-based decision-support system; DNN: deep neural network; EUS: endoscopic ultrasonography; GBC: gallbladder cancer; ML: machine learning; MRI: magnetic resonance imaging; SMI: superb microvascular imaging; US: ultrasonography; YOLOv3: You Only Look Once, version 3.

Study	Setting	Design/sample	Modality	Target	AI/comparator	Reference standard	Validation	Evidence stratum
Jeong et al. 2020 [9]	Korea	Retrospective; 535 total; temporal test n=98	Ultrasound	Neoplastic vs non-neoplastic polyps	Deep-learning decision support; reader assistance	Pathology	Temporal hold-out	A - Clinical DTA
Chen et al. 2020 [10]	China	Retrospective; n=224	High-resolution ultrasound	Neoplastic vs non-neoplastic polyps	Computer-aided diagnosis / machine learning	Pathology	Internal cross-validation	A - Clinical DTA
Yuan et al. 2020 [11]	China	Retrospective; 96 patients/99 lesions	Ultrasound	Adenoma vs cholesterol polyp	Radiomics	Pathology	Internal cross-validation	A - Clinical DTA
Kim et al. 2021 [12]	Korea	Retrospective multicenter; n=501	Ultrasound	True vs pseudopolyps	Ensemble CNN plus age/size	Pathology	Internal cross-validation	A - Clinical DTA
Jang et al. 2021 [13]	Korea	Retrospective; external cohort n=83	Endoscopic ultrasound	Neoplastic vs non-neoplastic polyps	EUS-AI	Pathology	External validation	A - Clinical DTA
Choi et al. 2023 [14]	Korea	Retrospective multicenter; n=263	Ultrasound	Neoplastic vs non-neoplastic polyps	Deep learning/CAD; reader assistance	Pathology	Internal cross-validation	A - Clinical DTA
Li et al. 2023a [15]	China	Retrospective multicenter; n=1,296	Ultrasound plus clinical data	Malignant potential in 8–15 mm polyps	Bayesian network	Pathology	Internal cross-validation	A - Clinical DTA
Li et al. 2023b [16]	China	Retrospective multicenter; n=759	Ultrasound plus clinical data	Neoplastic risk in >10 mm polyps	Bayesian network	Pathology	Internal hold-out	A - Clinical DTA
Yuan et al. 2023 [17]	China	Retrospective; n=100	B-mode plus superb microvascular imaging	Neoplastic vs cholesterol polyps	Dual-mode radiomics	Pathology	Internal cross-validation	A - Clinical DTA
Wang et al. 2023 [18]	China	Prospective multicenter; n=640	Ultrasound / contrast-enhanced ultrasound	Neoplastic and malignant masses	Radiomics machine learning	Pathology	Multiple internal test sets	A - Clinical DTA
Choi et al. 2026 [19]	Korea	Retrospective; small video validation cohort	EUS video	Neoplastic vs non-neoplastic polyps	EfficientNet video AI	Pathology	Video-level validation	A - Clinical DTA
Tang et al. 2026 [20]	China	Retrospective; n=339; independent test n=61	B-mode plus color Doppler	Neoplastic and malignant polyps	Integrative deep learning; reader assistance	Pathology	Independent test	A - Clinical DTA
Basu et al. 2023 [21]	India	Retrospective; n=218	Ultrasound	Gallbladder carcinoma	RadFormer	Pathology/curated labels	Internal cross-validation	A - Clinical DTA
Gupta et al. 2023 [22]	India	Prospective; n=565; temporal test n=273	Ultrasound	Gallbladder carcinoma	Deep-learning classifier vs radiologists	Clinical/pathologic reference	Temporal test	A - Clinical DTA
Xiang et al. 2024 [23]	China	Retrospective train/validation cohorts	Contrast-enhanced CT	Gallbladder carcinoma vs benign lesions	Deep learning vs radiomics/clinical models/radiologists	Pathology	Internal validation	A - Clinical DTA
Pang et al. 2019 [24]	China	Retrospective image dataset	CT	Cholelithiasis and stone type	YOLOv3 architecture	Image labels	Internal	B - Technical/non-clinical
Obaid et al. 2023 [25]	Multicountry dataset	Retrospective; 1,782 patients/10,692 images	Ultrasound	Nine gallbladder disease classes	Four deep neural networks	Dataset labels	Image-level split	B - Technical/non-clinical

Risk of Bias

No study was judged to be at low risk of bias across all the assessed domains. Retrospective, surgery-enriched recruitment was the most common concern. Internal validation, uncertain patient-level data separation, exclusion of technically difficult images, and incomplete threshold reporting further limited the generalizability of the findings. Prospective or independent testing in Jeong et al. [9], Wang et al. [18], Tang et al. [20], and Gupta et al. [22] reduced, but did not eliminate, concerns regarding spectrum bias and generalizability. Table 2 summarizes the risk-of-bias assessment.

**Table 2:** Risk-of-bias and applicability assessment of the included studies using a review-specific adaptation of QUADAS-2 Low indicates that the available information suggests that bias is unlikely or that there is low concern regarding applicability. High indicates that one or more methodological features are likely to introduce substantial bias or limit applicability to the review question. Unclear indicates that reporting was insufficient to permit a definitive judgment. Overall risk of bias was rated high when at least one risk-of-bias domain was high; unclear when no domain was high but at least one domain was unclear; and low only when all four risk-of-bias domains were low. Applicability concerns were assessed separately and did not contribute to the overall risk-of-bias judgment. The “Main source(s) of bias” column identifies only the principal methodological concern(s) for each study. QUADAS-2 was modified for this review by adding AI-specific signaling questions concerning patient-level versus image-level splitting, independence of training and test datasets, handling of multiple images per patient, prespecification of decision thresholds, exclusion of technically difficult cases, external validation, and potential data leakage. The modified tool was not formally validated as a separate instrument. QADAS: Quality Assessment of Diagnostic Accuracy Studies.

Study	Risk of bias: Patient selection	Risk of bias: Index test	Risk of bias: Reference standard	Risk of bias: Flow and timing	Applicability: Patient selection	Applicability: Index test	Applicability: Reference standard	Overall risk of bias	Main source(s) of bias
Jeong et al. 2020 [9]	High	Low	Low	Low	High	Low	Low	High	Retrospective surgery-enriched cohort, creating spectrum and selection bias.
Chen et al. 2020 [10]	High	High	Low	Unclear	High	Low	Low	High	Retrospective surgery-enriched cohort, internal validation, and unclear patient-level data splitting.
Yuan et al. 2020 [11]	High	High	Low	Unclear	High	Low	Low	High	Small lesion-level cohort, internal cross-validation, and possible within-patient correlation.
Kim et al. 2021 [12]	High	Unclear	Low	Unclear	High	Low	Low	High	Retrospective pathology-selected cohort and internal cross-validation.
Jang et al. 2021 [13]	High	Unclear	Low	Unclear	High	Low	Low	High	Retrospective selected cohort, small external test set, and incompletely reported threshold specification.
Choi et al. 2023 [14]	High	Unclear	Low	Low	High	Low	Low	High	Retrospective surgical cohort and internal cross-validation.
Li et al. 2023a [15]	High	Unclear	Low	Unclear	High	Low	Low	High	Retrospective surgical selection and internal model validation.
Li et al. 2023b [16]	High	Unclear	Low	Unclear	High	Low	Low	High	Retrospective surgical selection and internal model validation.
Yuan et al. 2023 [17]	High	High	Low	Unclear	High	Low	Low	High	Small single-center pilot cohort and internal cross-validation.
Wang et al. 2023 [18]	Unclear	Unclear	Low	Low	Unclear	Low	Low	Unclear	Pathology-confirmed selected mass cohort and restricted clinical spectrum.
Choi et al. 2026 [19]	High	High	Low	High	High	Low	Low	High	Small selected cohort and possible video- or image-level dependence or data leakage.
Tang et al. 2026 [20]	High	Unclear	Low	Low	High	Low	Low	High	Retrospective surgery-enriched selection and limited independent-test sample.
Basu et al. 2023 [21]	High	High	Unclear	Unclear	High	Unclear	High	High	Curated image dataset, internal cross-validation, and uncertain patient-level independence or reference labeling.
Gupta et al. 2023 [22]	Unclear	Low	Unclear	Low	Unclear	Low	Unclear	Unclear	Single-region temporal test cohort and incompletely described reference-standard composition.
Xiang et al. 2024 [23]	High	Unclear	Low	Unclear	High	Low	Low	High	Selected pathology-confirmed computed-tomography cohort and internal validation.
Pang et al. 2019 [24]	High	High	Unclear	High	High	High	High	High	Image-level technical dataset, internal cross-validation, and unclear clinical reference standard.
Obaid et al. 2023 [25]	High	High	High	High	High	High	High	High	Image-level splitting, dataset-label reference standard, possible patient leakage, and class-balancing effects.

Exploratory Descriptive Diagnostic Synthesis: Neoplastic Gallbladder Polyps

**Figure 2:**
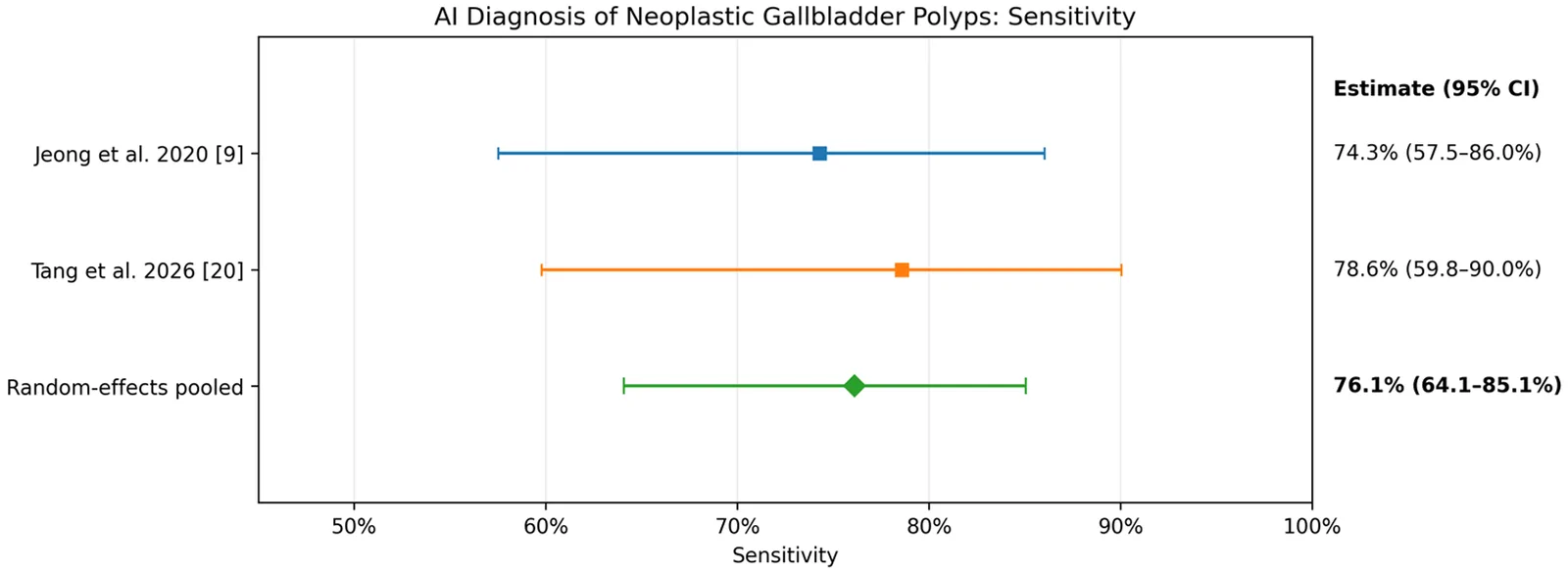
Individual-study sensitivity estimates and exploratory random-effects summary for neoplastic gallbladder-polyps Random-effects pooled sensitivity of AI for differentiating neoplastic from non-neoplastic gallbladder polyps in two independent or temporal test cohorts. Points indicate study estimates; horizontal lines indicate 95% confidence intervals.

**Figure 3:**
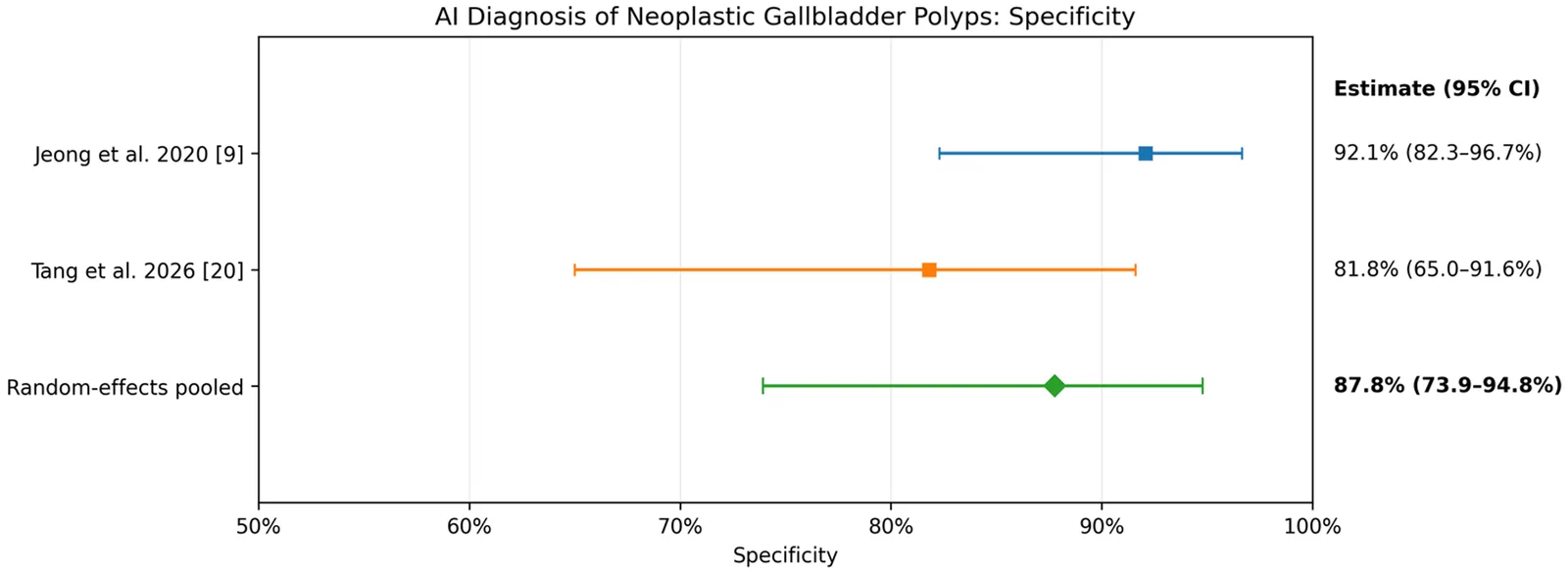
Individual-study specificity estimates and exploratory random-effects summary for neoplastic gallbladder-polyps Random-effects pooled specificity of AI for differentiating neoplastic from non-neoplastic gallbladder polyps in two independent or temporal test cohorts. Points indicate study estimates; horizontal lines indicate 95% confidence intervals.

Two independent or temporal validation cohorts provided reconstructable 2×2 diagnostic data, comprising 159 patients with 63 neoplastic and 96 non-neoplastic lesions. Jeong et al. [9] reported a sensitivity of 74.3% and specificity of 92.1%, whereas Tang et al. [20] reported a sensitivity of 78.6% and specificity of 81.8%. These individual-study estimates were considered the principal diagnostic evidence. Because only two eligible cohorts were available, formal bivariate random-effects or HSROC synthesis was not considered sufficiently reliable. Separate random-effects summaries were therefore calculated only for descriptive exploration, yielding a sensitivity of 76.1% (95% confidence interval (CI), 64.1%-85.1%; I²=0%) and specificity of 87.8% (95% CI, 73.9%-94.8%; I²=53.1%). The exploratory diagnostic odds ratio was 24.0 (95% CI, 10.1-57.1). Because separate pooling does not account for the within-study correlation or threshold relationship between sensitivity and specificity, these summary values should be regarded as provisional and hypothesis-generating rather than definitive joint DTA estimates. Table 3 presents the individual-study 2×2 results, with sensitivity and specificity estimates illustrated in Figures 2, 3.

**Table 3:** Individual-study diagnostic performance of AI for neoplastic versus non-neoplastic gallbladder polyps in independent or temporal validation cohorts Individual-study 2×2 results constitute the principal diagnostic evidence. Separate summary sensitivity and specificity estimates are presented only as descriptive exploratory analyses because only two eligible cohorts were available for synthesis. TP: True positive; FN: false negative; TN: true negative; FP: false positive.

Study	TP	FN	TN	FP	Sensitivity	Specificity	Validation
Jeong et al., 2020 [9]	26	9	58	5	74.3%	92.1%	Temporal hold-out
Tang et al., 2026 [20]	22	6	27	6	78.6%	81.8%	Independent test

Primary Diagnostic Meta-analysis: Neoplastic Gallbladder Polyps

The two eligible independent/temporal test cohorts included 159 patients, comprising 63 neoplastic and 96 non-neoplastic lesions. Random-effects pooled sensitivity was 76.1% (95% CI 64.1-85.1%; I²=0%). Pooled specificity was 87.8% (95% CI 73.9-94.8%; I²=53.1%). The pooled diagnostic odds ratio was 24.0 (95% CI 10.1-57.1; I²=0%). Because only two studies were available, these estimates should be interpreted as provisional and a bivariate HSROC model was not considered stable. The corresponding pooled sensitivity and specificity estimates are presented in Figures 2, 3, respectively.

Exploratory Accuracy Summary by Validation Design

**Figure 4:**
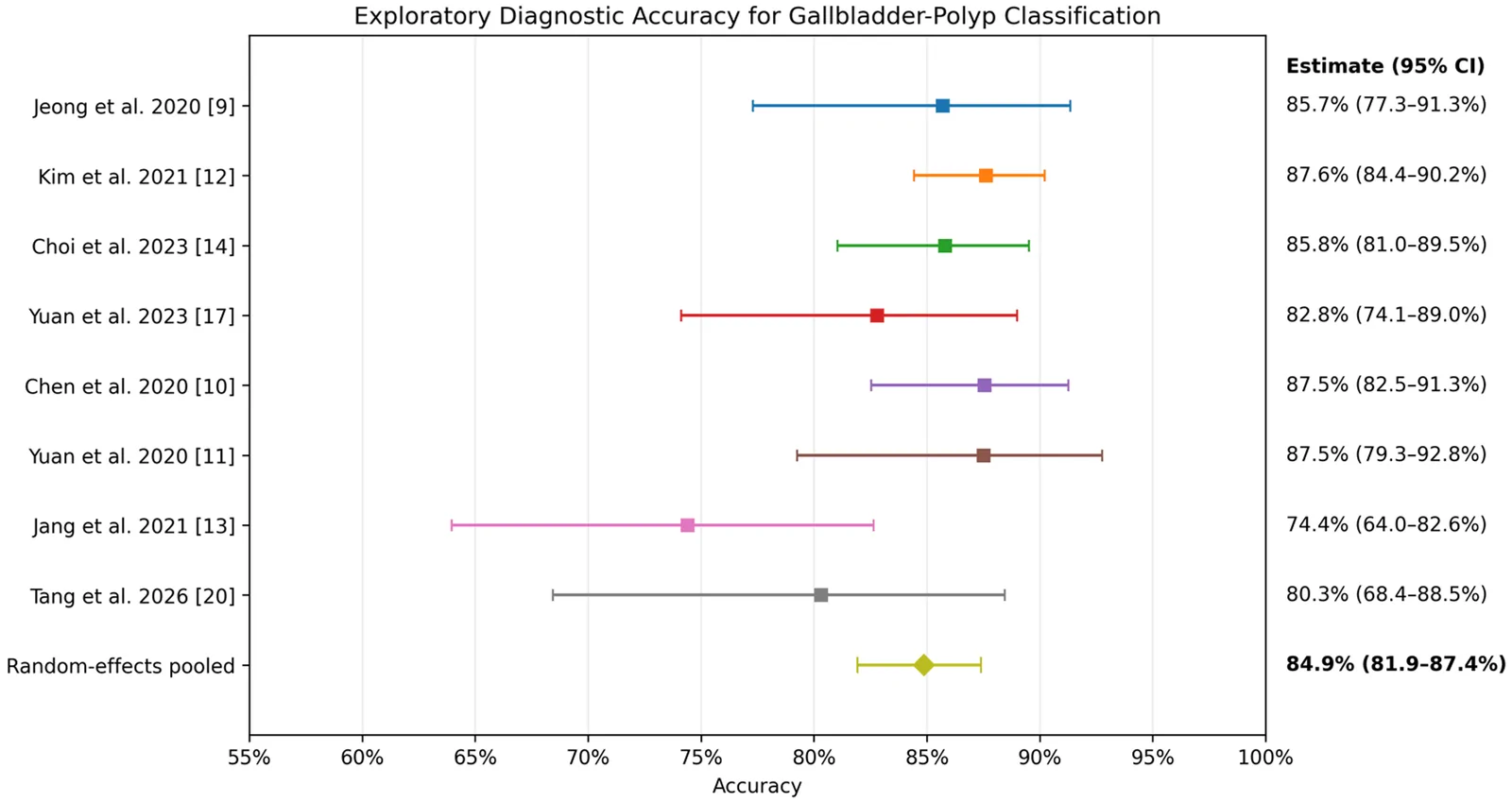
Exploratory random-effects meta-analysis of diagnostic accuracy for gallbladder-polyp classification. Points indicate study accuracy estimates; horizontal lines indicate 95% confidence intervals; the dashed vertical line indicates the pooled estimate.

Eight gallbladder-polyp studies reported overall accuracy with an interpretable patient- or lesion-level denominator. Because accuracy is dependent on prevalence, case mix, diagnostic threshold, target definition, and validation design, these results were treated as a secondary exploratory analysis rather than a primary diagnostic-performance estimate. The descriptive random-effects summary was 84.9% (95% CI, 81.9%-87.4%; I²=44.8%). Studies using external, temporal, or independent validation had a descriptive summary accuracy of 80.3% (95% CI, 72.5%-86.2%), compared with 86.7% (95% CI, 84.7%-88.5%) in internally validated studies. This difference should not be interpreted as a causal comparison because the groups also differed in target definition, case mix, modality, threshold selection, and study design. Most importantly, the 84.9% summary does not represent the expected clinical accuracy of AI when applied to a new patient population. Validation-specific individual-study results therefore provide the more clinically relevant interpretation (Table 4 and Figure 4).

**Table 4:** Exploratory descriptive accuracy summary of gallbladder-polyp studies by validation design Overall accuracy was summarized only as a secondary exploratory measure because it is influenced by prevalence, case mix, target definition, diagnostic threshold, and validation design. The reported summary estimates describe the included study samples and should not be interpreted as expected clinical accuracy in a new population. Comparisons between validation groups are descriptive and non-causal. CI: confidence interval; I²: percentage of total variability attributed to between-study heterogeneity.

Validation group and contributing studies	Number of studies	Pooled accuracy	95% CI	I²
All eligible gallbladder-polyp classification studies: Jeong et al. [9], Chen et al. [10], Yuan et al. 2020 [11], Kim et al. [12], Jang et al. [13], Choi et al. [14], Yuan et al. 2023 [17], and Tang et al. [20]	8	84.90%	81.9%-87.4%	44.80%
External, temporal, or independent validation: Jeong et al. [9], Jang et al. [13], and Tang et al. [20]	3	80.30%	72.5%-86.2%	44.10%
Internal cross-validation or hold-out validation: Chen et al. [10], Yuan et al. 2020 [11], Kim et al. [12], Choi et al. [14], and Yuan et al. 2023 [17]	5	86.70%	84.7%-88.5%	0%

AI-Assisted Versus Unaided Reader Interpretation

**Figure 5:**
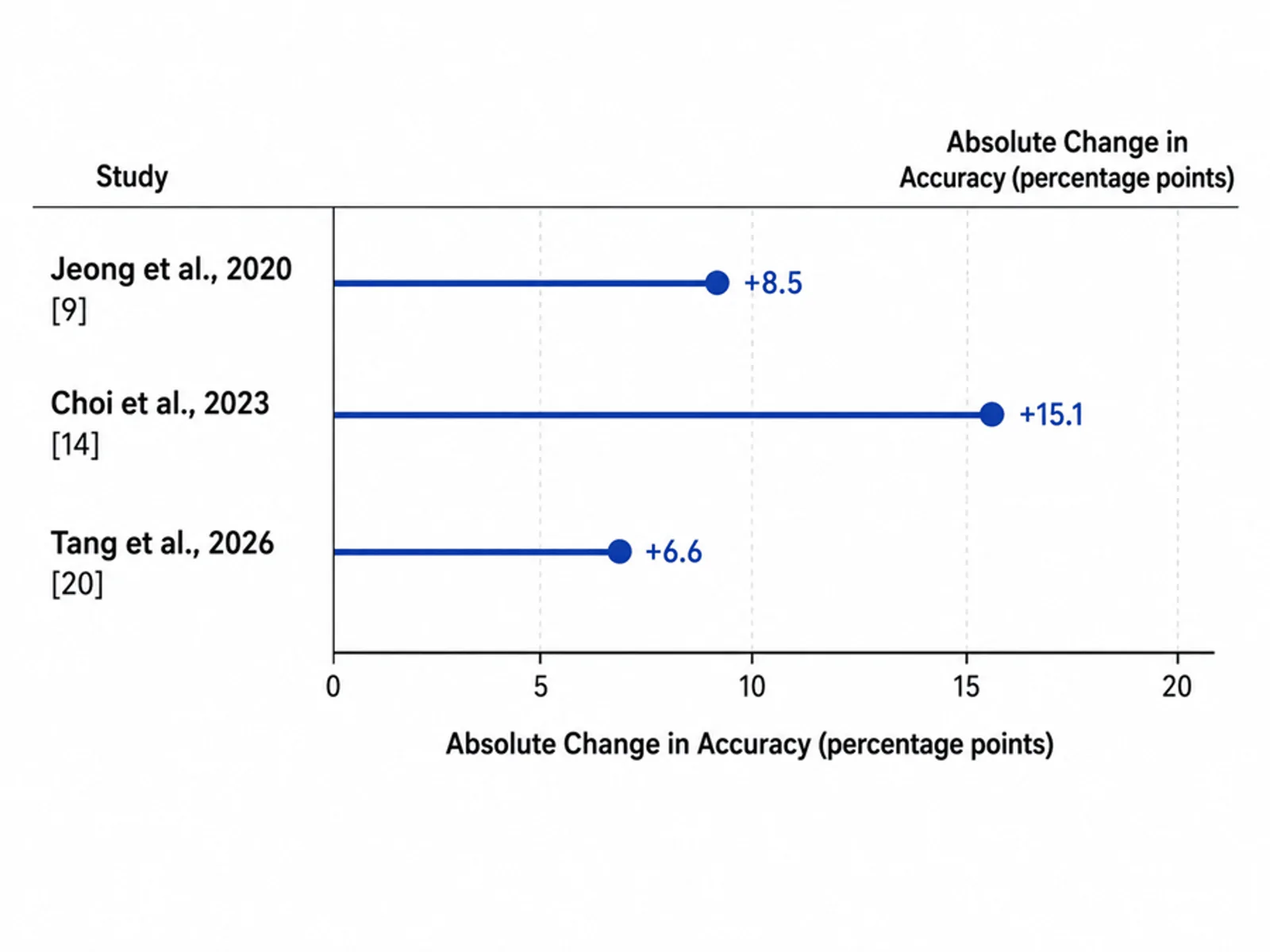
Study-specific absolute changes in reader accuracy with AI assistance Absolute changes in accuracy are shown descriptively for each study. No pooled effect estimate was calculated because paired case-level and reader-level variance information was unavailable.

Three studies directly evaluated reader performance with and without AI assistance. Because these studies used paired cases and, in some instances, repeated assessments by the same readers, formal pooling was not performed in the absence of paired or reader-level variance information. Jeong et al. [9] reported an increase in accuracy from 77.6% to 86.1%, corresponding to an absolute improvement of 8.5 percentage points. Choi et al. [14] reported an increase from 63.4% to 78.5%, an absolute improvement of 15.1 percentage points. Tang et al. [20] reported an increase from 78.7% to 85.2%, corresponding to an absolute improvement of 6.6 percentage points. Improvements tended to be larger among less-experienced readers in the individual studies, although differences in reader composition, study design, and reporting preclude a formal combined inference. Figure 5 shows the study-specific changes in reader accuracy with AI assistance compared with unaided interpretation. Table 5 summarizes the study-specific findings.

**Table 5:** Study-specific changes in reader accuracy with AI assistance compared with unaided interpretation Results are presented descriptively because the studies used paired or repeated-reader designs and did not consistently report the paired covariance, discordant-cell data, or reader-level variance required for valid inferential pooling. No pooled reader-assistance effect was calculated.

Study	Comparison unit	Unaided	AI-assisted	Difference	Interpretation
Jeong et al. 2020 [9]	Three radiologists; 294 ratings	77.60%	86.10%	+8.5 percentage points	The largest increase in area under the receiver operating characteristic curve was observed in the less-experienced reader (0.78 to 0.91).
Choi et al. 2023 [14]	Physician assessment; 263 cases	63.40%	78.50%	+15.1 percentage points	Inexperienced readers improved from 61.4% to 77.2%; the improvement in the experienced reader was not statistically significant.
Tang et al. 2026 [20]	Two junior radiologists; 122 ratings	78.70%	85.20%	+6.6 percentage points^a^	One junior reader showed a significant increase in area under the receiver operating characteristic curve; changes were smaller in the senior reader.

Gallbladder Carcinoma

The gallbladder carcinoma evidence was promising but heterogeneous. Gupta et al. reported prospective temporal-test sensitivity of 92.3%, specificity of 74.4%, and an AUC of 0.887 for ultrasound-based detection [22]. Basu et al. reported high internal cross-validation performance for a transformer-based model, but the curated dataset and absence of external testing created a high risk of optimism [21]. Xiang et al. reported the validation AUC of 0.857 for contrast-enhanced CT [23]. Wang et al. reported AUCs of 0.904-0.979 for carcinoma-versus-benign classification across prospective multicenter test sets, but uniform threshold-specific 2×2 data were unavailable [18]. Differences in modality, validation design, thresholds, and patient spectrum precluded pooling. Table 6 shows the diagnostic performance of artificial intelligence for gallbladder cancer across ultrasound and contrast-enhanced computed tomography studies.

**Table 6:** Diagnostic performance of artificial intelligence for gallbladder cancer across ultrasound and contrast-enhanced computed tomography studies The modality is presented in a separate column. A pooled estimate was not calculated because the studies differed in imaging modality, target definition, validation design, thresholds, and study populations

Study	Modality	Validation	AI performance	Conventional comparison	Interpretation
Gupta et al. 2023 [22]	Ultrasonography	Prospective temporal test; n=273	Sensitivity 92.3%; specificity 74.4%; AUC 0.887	Comparable to two experienced radiologists; the AI model showed higher sensitivity for wall-thickening-type gallbladder cancer than one reader	Strongest clinically oriented evidence, but based on a single-region test cohort.
Basu et al. 2023 [21]	Ultrasonography	Internal cross-validation; n=218	Accuracy 92.1%; sensitivity 92.3%; specificity 96.1%; AUC 0.971	Performance exceeded that of the reported radiologist comparator during internal cross-validation	High risk of optimistic performance because the dataset was curated and no external validation was performed.
Xiang et al. 2024 [23]	Contrast-enhanced computed tomography	Internal training and validation cohorts	AUC 0.864 in training and 0.857 in validation; validation sensitivity 85%	Compared with radiomics, clinical models, and three radiologists	Promising computed tomography evidence from a selected pathology-confirmed cohort.
Wang et al. 2023 [18]	Ultrasonography and contrast-enhanced ultrasonography	Prospective multicenter study; multiple test sets	Carcinoma-versus-benign AUC 0.904-0.979	Conventional ultrasonography AUC 0.706-0.766; ultrasound radiomics outperformed contrast-enhanced ultrasonography in one test set	Strong discrimination, but uniform reconstructable 2×2 threshold data were not reported.

Stratum B: Technical/Non-clinical Gallstone and Multiclass Image-classification Evidence

Two studies constituted Stratum B and were treated as technical/non-clinical evidence rather than clinical DTA evidence. Pang et al. developed a YOLOv3-based CT system for detecting cholelithiasis and classifying gallstone types [24]. Obaid et al. used 10,692 ultrasound images from 1,782 patients to classify nine gallbladder-disease categories, with MobileNet achieving 98.35% image-level accuracy [25]. These results were not pooled because image-level splitting, artificially balanced class distributions, limited clinical reference standards, and sparse reader comparisons produced a different estimand from patient-level clinical diagnosis. No robust patient-level comparative evidence was identified for acute or chronic cholecystitis. 

Publication Bias and Certainty of Evidence

Formal small-study-effect testing was not performed because the largest synthesis included eight studies and diagnostic-accuracy methods are unreliable in small heterogeneous samples. Selective reporting is a concern because many reports emphasized the best-performing model or cross-validation result and rarely reported calibration, failure analysis, decision curves, or all prespecified thresholds. Table 7 summarizes the qualitative confidence in the evidence across the 17 included studies, highlighting the size of the evidence base, the level of confidence for each outcome, and the main factors limiting certainty.

**Table 7:** Qualitative confidence across the clinical DTA and technical AI evidence strata Evidence domains were classified as Stratum A (clinically referenced diagnostic-accuracy evidence) or Stratum B (technical/non-clinical image-classification evidence) according to reference standard and unit of analysis. Stratum B evidence was not considered equivalent to patient-level clinical diagnostic evidence. Confidence ratings were based on evidence-base size, consistency, validation design, risk of bias, clinical applicability, and reporting completeness; these were not formal GRADE-DTA certainty ratings. GRADE: Grading of Recommendations, Assessment, Development, and Evaluation; DTA: Diagnostic test accuracy.

Outcome or evidence domain	Evidence base	Evidence stratum	Qualitative confidence	Primary reasons for limited confidence
Independent or temporal diagnostic performance for neoplastic gallbladder polyps	2 studies [9,20]	A - Clinically referenced DTA	Low	Only two eligible cohorts; small independent test samples; surgery-enriched patient selection.
Exploratory diagnostic accuracy for gallbladder-polyp classification	8 studies [9-14,17,20]	A - Clinically referenced DTA	Low	Accuracy was prevalence- and threshold-dependent; studies used mixed internal, external, and temporal validation methods.
AI-assisted reader interpretation	3 studies [9,14,20]	A - Clinically referenced DTA	Low	Few heterogeneous reader studies; paired and clustered data were unavailable for the meta-analysis.
Gallbladder cancer diagnosis	4 studies [18,21–23]	A - Clinically referenced DTA	Very low	Substantial heterogeneity in imaging modalities, diagnostic targets, reference standards, thresholds, populations, and validation designs.
Gallstones and multiclass gallbladder-disease classification	2 studies [24,25]	B - Technical/non-clinical	Very low	Predominantly image-level datasets, non-clinical dataset labels, possible data leakage, and limited comparison with clinical readers.

Discussion

This rapid evidence review indicates that artificial intelligence has potential for gallbladder imaging, particularly for characterization of gallbladder polyps and as an adjunct to clinician interpretation. However, the apparent performance of AI must be interpreted in the context of a small and methodologically heterogeneous evidence base. An important distinction in the revised review is between clinically referenced diagnostic accuracy evidence and technical/non-clinical image-classification evidence. Under this framework, 15 reports were considered clinically referenced studies, whereas two studies primarily evaluated classification against image or dataset labels. The latter provide evidence of technical discrimination within defined datasets but should not be regarded as equivalent to patient-level clinical diagnostic accuracy. The most clinically interpretable diagnostic evidence for neoplastic versus non-neoplastic gallbladder polyps came from only two independent or temporal validation cohorts with reconstructable 2×2 data. Jeong et al. [9] reported a sensitivity of 74.3% and specificity of 92.1%, whereas Tang et al. [20] reported a sensitivity of 78.6% and specificity of 81.8%. These individual-study estimates should be considered the principal diagnostic findings of the review. Because only two eligible cohorts were available, a bivariate random-effects or hierarchical summary ROC model could not be estimated with sufficient reliability. Separate random-effects summaries of sensitivity and specificity may provide a descriptive overview, but they do not preserve the within-study correlation between these measures or model the threshold-related trade-off. They should therefore be interpreted as exploratory and hypothesis-generating rather than as definitive pooled diagnostic test-accuracy estimates.

The original evidence base confirms that only these two independent or temporal cohorts provided reconstructable 2×2 data. Overall accuracy requires even greater caution. The eight gallbladder-polyp studies reporting accuracy differed in patient spectrum, prevalence, target definition, imaging approach, threshold selection, and validation strategy, including cross-validation, internal hold-out, temporal, external, and independent testing. Accuracy is inherently influenced by the proportion of diseased and non-diseased participants and therefore does not have a stable interpretation across populations with different case mixes. Accordingly, the previously calculated overall accuracy summary should be considered only a secondary exploratory sensitivity analysis. It cannot be interpreted as the expected clinical accuracy of an AI system when applied to a new population.

Greater weight should instead be given to validation-specific sensitivity, specificity, AUC, and individual-study findings. The apparent difference between internally validated and more independent validation studies is nevertheless clinically relevant as a signal of possible optimism in early model-development studies. Performance tended to be less favorable under external, temporal, or independent testing than under internal validation. This comparison should not be interpreted causally because validation category is confounded by differences in study design, case mix, diagnostic targets, modalities, thresholds, and sample size. Nevertheless, it reinforces the importance of testing locked AI models in populations that are genuinely independent of model development.

Patient-level separation is particularly important when multiple images or videos are obtained from the same participant, because image-level splitting can allow correlated observations from the same patient to appear in both development and evaluation datasets and thereby inflate apparent performance. The reader-assistance findings are encouraging but should also be interpreted descriptively. Three studies directly compared clinician performance with and without AI assistance. Jeong et al. [9] reported an absolute accuracy increase of 8.5 percentage points, Choi et al. [14] reported an increase of 15.1 percentage points, and Tang et al. [20] reported an increase of 6.6 percentage points. These studies used paired cases and, in some instances, repeated assessments by the same readers. Because discordant-pair data, within-reader covariance estimates, and reader-level variance information were not consistently available, a statistically valid paired or hierarchical meta-analysis could not be performed. The study-specific changes were therefore retained without an inferential pooled estimate. The observation that larger improvements were sometimes seen among less-experienced readers is potentially important, but the available evidence is insufficient to establish whether AI consistently produces greater benefit according to reader experience. The most defensible present role for these systems is therefore as decision-support, second-reader, educational, or quality-assurance tools rather than replacements for clinical interpretation.

Evidence for gallbladder carcinoma was promising but too heterogeneous for meaningful quantitative synthesis. Gupta et al. [22] reported temporal-test sensitivity of 92.3%, specificity of 74.4%, and an AUC of 0.887 for ultrasound-based detection. Wang et al. [18] reported AUC values of 0.904-0.979 across prospective multicenter test sets, whereas Xiang et al. [23] reported a validation AUC of 0.857 for contrast-enhanced computed tomography. Basu et al. [21] reported strong internal performance, although the curated dataset and absence of external validation raise concern regarding optimism. Differences in modality, target definition, reference standard, threshold, patient spectrum, and validation design prevented a common pooled estimate. Prospective and multicenter findings are encouraging, but stronger evidence is needed before these models can be considered generalizable to routine gallbladder-cancer diagnosis. The distinction between clinical and technical evidence is particularly relevant for gallstones and multiclass gallbladder-disease classification. Pang et al. [24] evaluated a CT-based system for cholelithiasis and stone classification, whereas Obaid et al. [25] evaluated deep-learning classification using 10,692 ultrasound images from 1,782 patients. However, image-level splitting, dataset-label reference standards, class balancing, and limited clinical-reader comparison result in a different estimand from patient-level diagnostic performance. Consequently, high technical classification accuracy in these datasets should not be interpreted as evidence that equivalent performance would occur in unselected patients encountered in routine care.

Robust clinically referenced evidence for cholecystitis and several other common gallbladder disorders remains particularly limited. Several methodological issues also reduce confidence in the current evidence. Many studies were retrospective and derived from surgery- or pathology-enriched cohorts. Such populations preferentially include larger, more suspicious, or otherwise selected lesions and may not reflect the broader spectrum of gallbladder abnormalities encountered during routine ultrasonography. Risk-of-bias concerns also included internal validation, uncertain patient-level separation, exclusion of technically difficult images, incomplete threshold reporting, and possible data leakage. The manuscript's risk-of-bias assessment consequently identified substantial concerns across much of the evidence base. These limitations emphasize the need to distinguish model-development performance from evidence of clinical effectiveness. Future studies should therefore begin with a clearly defined intended clinical use.

Models intended to distinguish neoplastic from non-neoplastic polyps, support malignancy referral, assist novice readers, or triage uncertain examinations require different study designs and outcome measures. Development datasets should use strict patient-level separation, and final models, preprocessing steps, and thresholds should be locked before independent testing. External validation should include different institutions, geographic regions, scanner manufacturers, operators, and clinically representative populations. Calibration, uncertainty, failure cases, subgroup performance, and the handling of technically inadequate examinations should be reported routinely. For reader-assistance systems, prospective multireader multicase studies should preserve paired case-level and reader-level data so that appropriate hierarchical analyses can be performed. Ultimately, demonstrating diagnostic discrimination alone is insufficient to establish clinical utility. Future studies should determine whether AI changes management decisions and improves outcomes that matter to patients and health systems, including unnecessary cholecystectomy, missed malignancy, additional investigations, referral decisions, interpretation time, cost-effectiveness, and downstream patient outcomes. Human-AI studies should also evaluate automation bias and establish explicit procedures for situations in which AI output conflicts with clinician judgment.

Limitations of the Review

This review itself has important limitations. The evidence-identification process used a previously published scoping review as an anchor followed by a targeted PubMed update and supplementary publisher-website, reference-list, and citation searching rather than an independent comprehensive search of multiple bibliographic databases. Complete record-level logs were also unavailable for some components of the historical and supplementary searches; therefore, exact identification, deduplication, screening, and retrieval counts could not be reconstructed reliably. The review should consequently be interpreted as a rapid evidence review rather than an exhaustive systematic search.

Screening, extraction, and risk-of-bias assessment were not conducted independently in duplicate, and the review-specific adaptation of QUADAS-2 was not prospectively registered as a separate instrument. The modified signaling questions and decision rules should therefore be reported transparently in the supplement together with study-level supporting evidence. In addition, only two independent or temporal cohorts provided reconstructable 2×2 data, preventing reliable joint bivariate or HSROC synthesis. Overall accuracy was available from heterogeneous studies and is prevalence-, case-mix-, and threshold-dependent; any accuracy summary is therefore exploratory and cannot predict performance in a new clinical population. Paired case-level and reader-level covariance information was unavailable for the reader-assistance studies, preventing valid inferential pooling. These limitations, together with heterogeneous modalities, targets, thresholds, reference standards, and validation designs, substantially constrain the certainty and generalizability of the findings. The original manuscript similarly acknowledges the targeted search, lack of duplicate review procedures, limited independent cohorts, missing paired-reader information, and heterogeneous reference standards.

## Conclusions

The available evidence suggests that AI has potential in gallbladder imaging, but the strength of evidence differs substantially according to study design and reference standard. The revised evidence framework distinguishes 15 clinically referenced diagnostic studies from two technical/non-clinical image-classification studies, and these groups should not be interpreted as providing equivalent evidence of clinical diagnostic performance. For neoplastic gallbladder-polyp classification, the strongest directly interpretable evidence comes from only two independent or temporal validation cohorts. Individual-study sensitivities ranged from 74.3% to 78.6%, and specificities ranged from 81.8% to 92.1%. These results are encouraging but are insufficient to establish a definitive pooled diagnostic-accuracy estimate. Separate summaries of sensitivity and specificity remain descriptive because the evidence base is too small for reliable bivariate or HSROC modeling. Three reader-assistance studies also reported improvements in accuracy with AI support, with study-specific absolute gains of 6.6-15.1 percentage points. These results were not pooled because appropriate paired case-level and reader-level variance data were unavailable. They therefore support the potential value of AI as a second reader, decision-support system, or quality-assurance tool, particularly as an aid to less-experienced clinicians but do not establish a definitive pooled reader-assistance effect. Overall accuracy across heterogeneous studies should not be interpreted as the expected performance of AI in a new clinical population. Similarly, technically strong image-classification results based on dataset or image labels do not establish patient-level clinical diagnostic accuracy. Evidence for gallbladder carcinoma is promising but heterogeneous, while clinically referenced evidence for gallstones, cholecystitis, and other common gallbladder disorders remains limited. Routine or autonomous clinical implementation is therefore premature. Prospective multicenter studies using locked models, independent patient-level validation, prespecified thresholds, calibrated outputs, representative clinical populations, and appropriately designed paired-reader analyses are required. Most importantly, future studies should demonstrate that AI improves clinical decision-making and patient-relevant outcomes rather than diagnostic performance metrics alone.
